# Supplementary material for: Structural Stabilization of Human Transthyretin by Centella asiatica (L.) Urban Extract: Implications for TTR Amyloidosis
Source: Biomolecules. 2019 Mar 29;9(4):128. doi: 10.3390/biom9040128 (PMC6523946; doi:10.3390/biom9040128)
Supplement: Supplementary file 1 [file biomolecules-09-00128-s001.pdf]

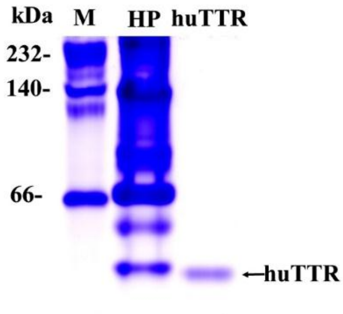

(a)

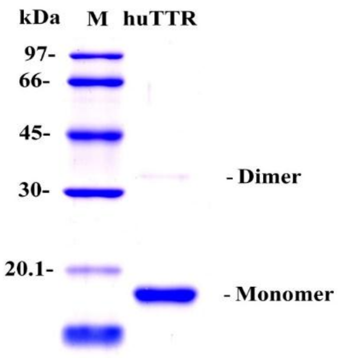

(b)

Figure S1. Purification of human transthyretin (huTTR) from human plasma with reduced albumin burden (HP). Native-PAGE (**a**) and SDS-PAGE (**b**) analysis of the purified fraction of huTTR after preparative discontinuous native-PAGE. M: Protein molecular weight markers.

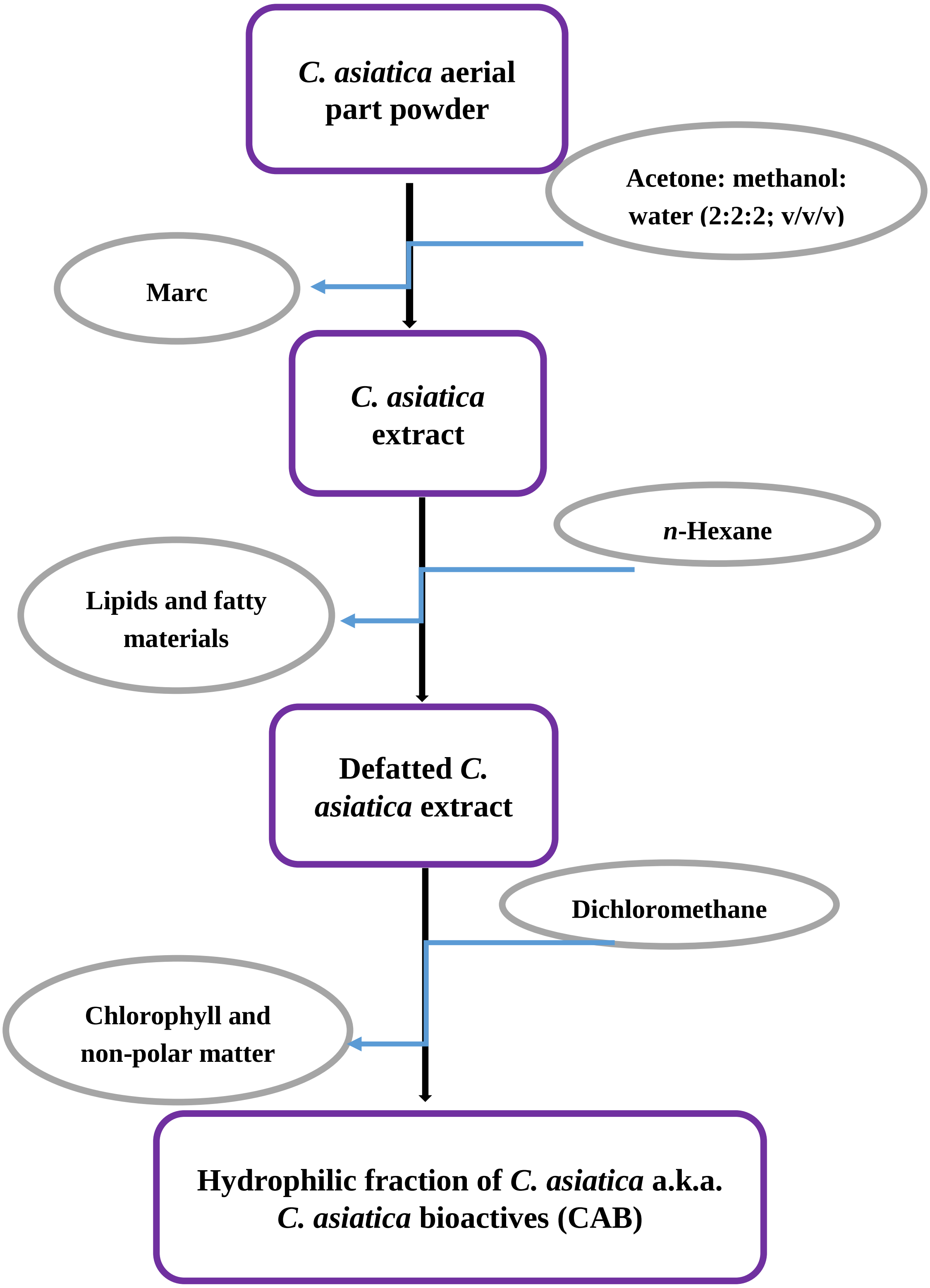

Figure 2. Scheme representing the preparation of CAB from *C. asiatica*.

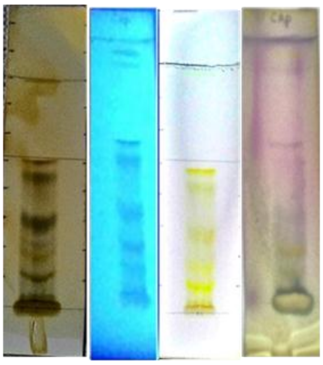

(a) (b) (c) (d)

Figure 3. Thin layer chromatography profile of CAB. (**a**) TLC plate dipped in 3% ferric chloride reagent for 1 second and heated at 100 ^{0}C for 7 minutes. (**b**) Underivatized plate viewed under 254 nm ultraviolet light. (**c**) TLC plate saturated with 28% ammonia solution and sprayed with 2% AlCl3 in methanol solution. (**d**) TLC plates dipped in Anisaldehyde-sulfuric acid reagent for 1 second and heated at 105 ^{0}C for 10 minutes.

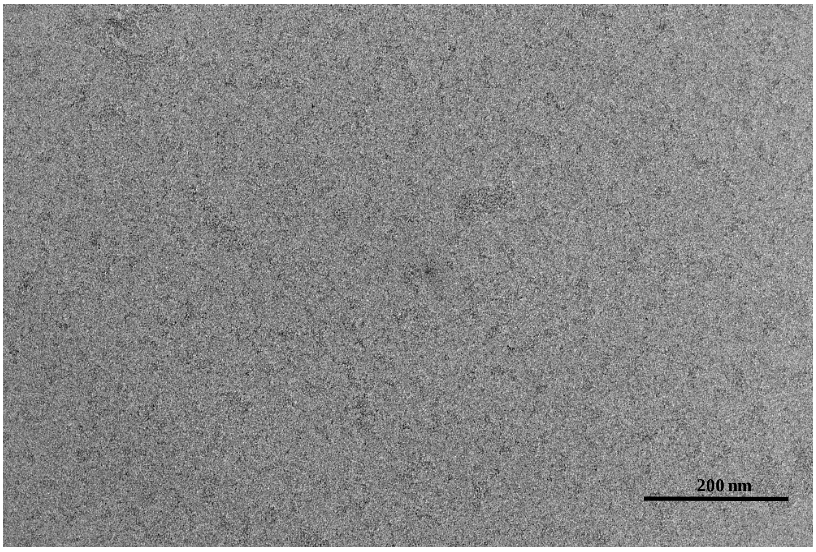

Figure 4. TEM image of CAB alone, in the absence of huTTR, incubated at pH 4.0 and 37 ^{0}C, for 7 days. Scale bar represents 200 nm.
